# Mechanism of action of glycyrrhizin against *Plasmodium falciparum*

**DOI:** 10.1590/0074-02760210084

**Published:** 2021-08-20

**Authors:** Maria de Nazaré Correia Soeiro, Gérard Vergoten, Christian Bailly

**Affiliations:** 1 Fundação Oswaldo Cruz-Fiocruz, Instituto Oswaldo Cruz, Laboratório de Biologia Celular, Rio de Janeiro, RJ, Brasil; 2 University of Lille, Inserm, Institut de Chimie Pharmaceutique Albert Lespagnol, Faculté de Pharmacie, Lille, France; 3 OncoWitan, Lille, France

**Keywords:** cholesterol, glycyrrhizin, glyoxalase 1, HMGB1, Malaria, Plasmodium

## Abstract

Extracts of the plant *Glycyrrhiza glabra* (licorice) are used in traditional medicine to treat malaria. The main active components are the saponin glycyrrhizin (GLR) and its active metabolite glycyrrhetinic acid (GA) which both display activities against *Plasmodium falciparum*. We have identified three main mechanisms at the origin of their anti-plasmodial activity: (i) drug-induced disorganisation of membrane lipid rafts, (ii) blockade of the alarmin protein HMGB1 and (iii) potential inhibition of the detoxifying enzyme glyoxalase 1 (GLO-1) considered as an important drug target for malaria. Our analysis shed light on the mechanism of action of GLR against *P. falciparum*.

Malaria, caused by protozoa parasites belonging to the genus *Plasmodium*, remains an important threat to public health worldwide. Different *Plasmodium* species have been implicated in the disease, such as *P. falciparum*, *P. vivax*, *P. malariae*, *P. ovale* and *P. knowlesi* but the mortality from malaria is essentially due to infections with *P. falciparum*. This vector borne disease is endemic in more than 90 countries, affecting approximately 40% of the world’s population. Up to now, no single drug can eliminate all parasite forms, and therapy depends on geographic area, *Plasmodium* species, clinical outcomes, and disease severity, usually including more than one drug classes, with independent modes of action, given simultaneously. Quinoline-like compounds (such as the cinchona alkaloid quinine, 4-aminoquinoline chloroquine (CQ), hydroxychloroquine, primaquine, pyronaridine) and artemisinin-based combination therapies are used to treat the disease. But drug-resistances have emerged possibly related to mutations in the active sites of drug targets or from the biochemical changes in the drug receptors, and thus novel approaches are necessary.

The effectiveness of the two plant-based antimalarial drugs, quinine and artemisinin, encouraged the search for antimalarial agents from diverse botanic sources. *Glycyrrhiza glabra*, also known as licorice, is an easily cultivated small perennial herb that represents one of the most important medicinal plants, largely used in traditional herbal formulas to treat a variety of diseases and conditions. This herb displays antioxidant, antifungal, anticarcinogenic, anti-inﬂammatory, and cytotoxic properties. As they act in febrile cases, extracts of *G. glabra* are used in traditional medicine to treat malaria in different countries*,* representing one of the main anti-malarial ancient Iranian arsenal.^1^

Extracts and chloroformic fractions from Iran botanic sources of aerial part of *G. glabra* have shown marked antiplasmodial activity and a selectivity for *P. falciparum* and *P. berghei in vitro* (IC_50_: 8.9 μg/mL against the chloroquine-resistant K1 strain with a CHCl_3_ extract but IC_50_ > 64 μg/mL on the 3D7 CQ-sensitive parasite strain). Also, a significant *in vivo* activity has been reported (86 % suppression of parasitaemia at 10 mg/kg in mice, with the CHCl_3_ extract).^2^ More recently, another study demonstrated a marked *in vitro* and *in vivo* activity of different fractions of root extracts of *G. glabra* collected from the Tarom district of Zanjan Province (Iran). Water-methanol and ethyl acetate fractions showed high potency *in vitro* against the 3D7 strain (IC_50_ = 9.95 and 13 µg/mL, respectively), with selectivity indices higher for the water-methanol fraction. Both samples reduced animal parasitaemia (72.2% and 65%, respectively) in mice experimentally infected with *P. berghei*.^3^

These findings corroborate previous works. Sangian and collaborators^4^ reported the antiplasmodial activity of hydroalcoholic extracts from roots *of G. glabra* L. (Papilionaceae). They evidenced a promising activity *in vitro* using the 3DJ strain (IC_50_ = 13.56 μg/mL), with mild toxicity against brine shrimp *Artemia salina* larvae, leading to a moderate selectivity index (SI = 23). The crude extracts (400 mg/kg) partially reduced (65%) parasitaemia of mice infected with *P. berghei*, while CQ completely suppressed the parasite. Similar effects were found for ethyl acetate extracts from *G. glabra* roots. The analysis upon *P. falciparum* (CQ-sensitive and resistant 3D7 and INDO strains, respectively) showed a considerable anti-plasmodial activity of the extracts *in vitro* (IC₅₀ = 6 and 4.5 μg/mL, respectively). The authors suggested that the roots may be a better source for anti-plasmodial molecules as compared to the aerial part of the plant.^5^

These pre-clinical studies support the traditional use of *G. glabra* to treat malaria, but the origin of the activity is unclear because the extracts usually contain a large diversity of bioactive molecules, including triterpenoid saponins, coumarin and flavonoids. A constituent of the roots of *G. glabra*, the polyphenolic flavonoid glabridin, displays an anti-plasmodial activity upon *P. falciparum in vitro*, exhibiting a noticeable activity (IC_50_ = 23.9 μM), besides a poor cytotoxicity to Vero cell line (IC_50_ = 246 μM) and a reasonable SI (SI = 9.6).^6^ However, the most abundant component of *G. glabra* (and liquorice preparations in general) is the monodesmosic saponin glycyrrhizin (GLR). Its main metabolite glycyrrhetinic acid (GA) has been evidenced as a potent antimalarial agent, active *in vitro* against *P. falciparum* (IC_50_ = 1.69μg/mL upon NF54 strain). A dose-dependent *in vivo* activity of this GLR metabolite was also reported, with a complete extinction of the parasitaemia in mice infected with *P. berghei* K173 strain (sensitive to CQ), at the GA dose of 250mg/kg, but with cure rates lower than CQ.^7^

Therefore, GLR and/or its metabolites represent interesting antimalarial agents, worth of further investigations against different species of *Plasmodium*. But their molecular mechanism of action is unclear at present. This is the reason why we have analysed the potential targets and pathways implicated in the antimalarial activity of GLR. Here, we present and discuss the different pharmacological activities of GLR and its two main metabolites - the mono-glycoside MGA and the aglycone GA (Fig. 1) - that could contribute to the anti-malarial activity. A better understanding of the anti-malarial activity of GLR will help and guide the design of new drugs.

**Fig. 1:**
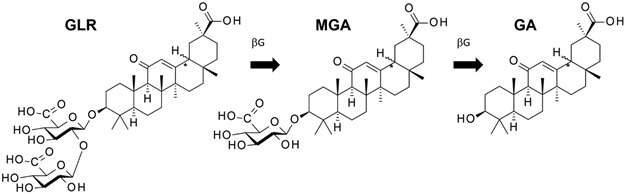
structures of glycyrrhizin (GLR) and its plasma metabolites 3-O-mono-β-D-glucuronyl-glycyrrhetinic acid (MGA) and glycyrrhetinic acid (GA). The parent product bearing a disaccharide is converted into the monosaccharide MGA and the aglycone GA under the action of β-glucuronidases (βG).

*GLR-induced sequestration of cholesterol and disorganisation of lipid rafts* - *P. falciparum* needs cholesterol but lacks a cholesterol synthesis pathway. The pathogen must use host cholesterol for its metabolism, possibly explaining why malaria patients generally have a low blood cholesterol level. *P. falciparum* can import free cholesterol or inner erythrocytic membrane-derived cholesterol. The *Plasmodium* infection remodels human erythrocytes with new membrane systems and the cholesterol level affects membrane trafficking. For example, *P. vivax* depletes cholesterol from the *P. vivax*-infected reticulocyte cell membrane.^8^ The cholesterol dependency of the parasite can be exploited for drug delivery, using cholesterol-drug conjugates, thereby increasing the antimalarial activity of the payload delivered to the infected cells via cholesterol transporters. Interestingly, it has been shown that cholesterol sequestration can reduce *P. berghei* liver-stage parasites burden *in vivo*.^9^

GLR, like other saponins, is an amphipathic glycoside capable of penetrating membranes, with a high affinity for cholesterol. Saponins have been used for a long time for permeabilising cholesterol-rich mammalian cells, including erythrocytes and the reduction of membrane cholesterol by the parasites can induce resistance of erythrocytes to saponins.^10^ Saponins reduce the movement of molecules within the membrane and thus impede the formation of fusion vesicles necessary for the entry of the parasite. However, it is important to underline that GLR is a non-lytic saponin. In contrast to other saponins like digitonin, GLR shows weak permeabilising effects and has very low hemolytic profile.^11^ It does not destroy the whole membrane integrity and induces little leakage from liposomes compared to other saponins.^12^ GLR interacts with cholesterol and this interaction modifies the membrane permeability to ions and small molecules. In a system of artificial membranes prepared at 1.5:1 cholesterol-to-phospholipid mole ratio, it was shown that GLR reduced cholesterol domain formation by 54.9%.^13^ Therefore, it can be argued that at least a part of the membrane effects of GLR can be linked to an interaction with cholesterol and the reduction in cholesterol domain in membrane. As illustrated in Fig. 2, GLR can form stable complexes with cholesterol, mainly via hydrophobic interaction with the sapogenin moiety of GLR. The potential energy of interaction is equivalent for GLR and MGA bound to cholesterol. The energy of the GLR-cholesterol complex (ΔE = -21.5 kcal/mol) is more negative compared to that measured with GA (ΔE = -17.1 kcal/mol), indicating that there is also a contribution from the carbohydrate moiety of GLR/GA in the interaction with cholesterol. The interaction energy is not extremely high (in agreement with the very weak hemolytic capacity of GLR), however, the interaction is significant and sufficient to induce plasma membrane perturbations.

**Fig. 2:**
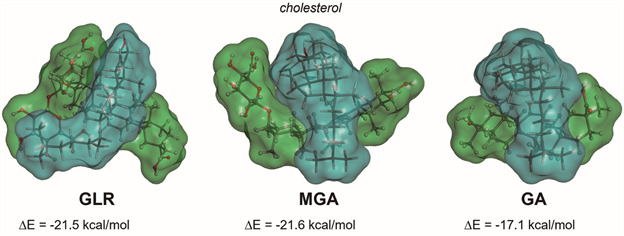
molecular models of the interaction between the indicated compound (green) and cholesterol (blue). Complexes are stabilised by stacking interactions between the hydrophobic part of each molecule and with glycyrrhizin (GLR) and 3-O-mono-β-D-glucuronyl-glycyrrhetinic acid (MGA), additional contacts implicating the glycoside moiety contribute to clamp the cholesterol unit. In each case, the computed potential energy of interaction (ΔE) is indicated.

In addition, the trapping of cholesterol by GLR concurs to a drug-induced destabilising effect on lipid rafts, which are membrane microdomains involved in the entry of the pathogen. These rafts, enriched in cholesterol and sphingolipids, form highly dynamic systems implicated in the erythrocyte invasion process by *P. falciparum*. The parasite induces a cytoplasmic remodeling of erythrocyte raft lipids during infection and the disruption of the lipid rafts by drugs like lidocaine can inhibit the parasitic invasion.^14^ Not only GLR decreases the level of cholesterol in lipid rafts but also inhibits the translocation of proteins to lipid rafts. This interaction contributes to the regulation of the size of lipid raft domains observed in the presence of GLR.^15^ But cholesterol is not the only membrane component implicated in the drug-induced perturbation of rafts. GA was found to interact even more strongly with a raft monolayer model than GLR and the raft domains became smaller as the concentrations of GA in the subphase increased.^15^

*GLR binding to PfHMGB1 protein* - As it is frequently the case with natural products, GLR has multiple molecular targets in addition to interacting with membrane cholesterol. At least eight potential protein targets have been mentioned with GLR, MGA or GA, but only three of these eight protein targets are possibly relevant to the anti-malarial activity of the drug: HMGB1, EGFR and Glyoxalase 1.^16^ ^,^ ^17^ ^,^ ^18^ The other targets evoked in different studies are not relevant to malaria [such as the enzymes 11β-hydroxysteroid dehydrogenase (11β-HSD), kynurenine aminotransferase 2 (KAT2), butyrylcholinesterase, β-site amyloid precursor protein cleaving enzyme 1 (BACE1) and the Kelch like ECH-associated protein 1 (Keap 1)].

The link between GLR, *Plasmodium* and epidermal growth factor receptor (EGFR) is weak. On the one hand, it has been reported that the anticancer activity of MGA observed with xenograft tumor mouse models could be linked to the capacity of the drug to interact with EGFR, proposed on the basis of a molecular modeling but there is no experimental validation of this *in silico* proposal.^17^ On the other hand, small kinase inhibitors capable of blocking EGFR and displaying an antimalarial activity have been described. But the best antimalarial compounds in these series were inactive against EGFR; thus, the two aspects are likely not connected. The compounds likely target another kinase in the parasite.

In contrast, the link between GLR, *Plasmodium* and HMGB1 (high-mobility group box 1) is much stronger and interesting. GLR is certainly the best characterised binder to human HMGB1 which is an abundant and highly conserved protein. Nuclear HMGB1 is a DNA chaperone and protector from apoptotic cell death. Cytoplasmic and extracellular HMGB1 is a prototypic damage-associated molecular pattern molecule (DAMP) also known as alarmins, which orchestrates the inflammatory and immune response. As an alarmin, extracellular HMGB1 activates innate immunity via the binding to different receptors, such as RAGE, TLR-2/4 and CXCR-4. Its multi-facet functions make HMGB1 an interesting therapeutic target for the treatment of various diseases, including cancer, neurodegenerative disorders, sepsis and other inflammatory diseases. HMGB1 is also important in the context of *Plasmodium* infections for two main reasons. First, HMGB1 is an important mediator of inflammation and the systemic release of the protein is associated with severe and fatal outcome in patients with *P. falciparum* infection. Parasitised erythrocytes induce HMGB1 release from human peripheral blood mononuclear cells *in vitro* and the neutralisation with antibodies directed against HMGB1 reduces mortality in a murine model of severe malaria.^19^ The link has also been evidenced in human. Sera from patients with severe and uncomplicated malaria have significantly higher circulating HMGB1 levels compared with healthy controls.^20^ The sequestration of *P. falciparum*-infected erythrocytes in the renal microcirculation leads to renal damages which then lead to obstruction, hypoxia and ischemia.^21^ Human HMGB1 is thus a potential target for therapeutic intervention, to reduce the damages caused by the parasite.

Second and most importantly, *P. falciparum* encodes its own HMGB1 (*Pf*HMGB1), with a single HMG box very similar to the HMG Box-B of Human HMGB1. A recent analysis of the 71-amino acid sequence of *Pf*HMGB1 has shown that it exhibits 39.4 % identity and 54.9% similarity with *Hs*HMGB1.^22^ In Human, the two-boxes protein HMGB1 has dual cytokine activities, with the Box-A viewed as the anti-inflammatory box and Box-B considered as the pro-inflammatory box. In *Plasmodium*, the one-box protein analogous to human Box-B has pro-inflammatory functions, inducing pro-inflammatory cytokines such as TNFα from mouse peritoneal macrophages.^23^

Like *Hs*HMGB1, *Pf*HMGB1 can interact with distorted DNA structures and bend linear DNA. The protein is preferentially expressed in asexual erythrocytic stages of the parasite and likely plays a role in the transcriptional regulation of *Plasmodium* development in erythrocytes. Recently, DNA aptamers specifically recognising *Pf*HMGB1, not *Hs*HMGB1, have been identified and proposed for the rapid detection of *P. falciparum* infection.^22^

The tridimensional structure of the HMGB1 protein from *P. falciparum* 3D7 strain has been solved by NMR. It is a 91-amino acid protein (expressed in *Escherichia coli*) that presents three helices very similar to those observed with Box-B of *Hs*HMGB1. The binding site for GLR in the human protein is known because the structure of the protein-drug complex has been solved by NMR^16^ and we have recently analysed further this complex by molecular modeling.^24^ We have established molecular models for the binding of GLR, MGA and GA to *Pf*HMGB1 to compare the binding capacity of the products to *Pf*HMGB1 versus *Hs*HMGB1 (Fig. 3). The binding energies calculated with *Pf*HMGB1 and *Hs*HMGB1 are comparable (Table). There is no doubt that GLR can bind well to the HMGB1 protein from the parasite, perhaps even better than to the Human HMG box. The amino acid contacts are different but, in both cases, the drug engages interactions with the HMG protein both via its triterpenoid core and its carbohydrate moiety. As with Human HMGB1, the disaccharide moiety of GLR contributes importantly to the interaction with the HMGB1 protein from the parasite. Both glucuronic acid residues of GLR are involved in the protein interaction. The removal of one of glucuronic acid residue decreases the extent of binding; the removal of the two glucuronic acid residues (to afford GA) reduces the capacity of binding by about 50%, similar to what has been observed with the Human protein.^24^ It will be necessary to validate the proposed interaction experimentally, but it seems clear that *Pf*HMGB1 represents a bona fide target for GLR.

**Fig. 3:**
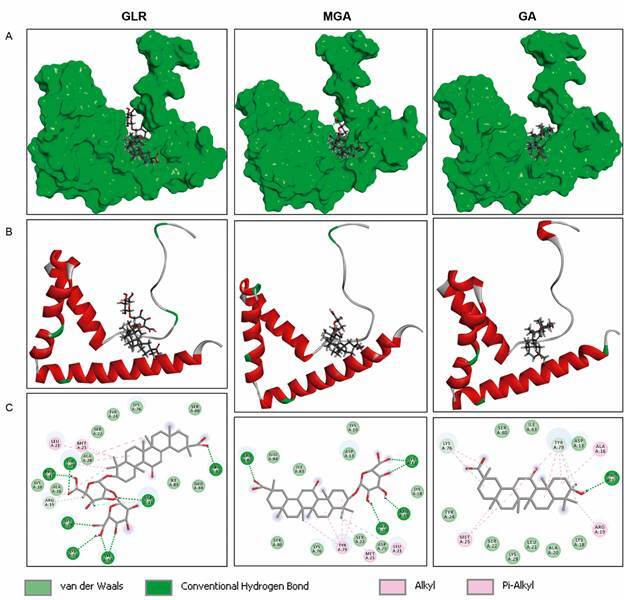
molecular models of the interaction of glycyrrhizin (GLR), 3-O-mono-β-D-glucuronyl-glycyrrhetinic acid (MGA) and glycyrrhetinic acid (GA) with HMG1 from *Plasmodium falciparum* (PDB code: 2MRC). The procedure used to construct the models is described Table. In all cases, the drugs correspond to the 18α-epimers. The protein surface is shown in green (A) or a ribbon model in red (B) to illustrate the three helices. (C) Binding map contacts are shown, with the indicated colour code.

**TABLE:** Calculated potential energy of interaction (ΔE) and free energy of hydration (ΔG) of the drug-HMGB1 complexes^*a*^

	*Pf* HMGB1^*b*^		*Hs* HMGB1 (Box-B)^*b*^	
Cpds	ΔE (kcal/mol)	ΔG (kcal/mol)	ΔE (kcal/mol)	ΔG (kcal/mol)
GLR	-96.57	-32.70	-83.66	-21.65
MGA	-79.73	-29.50	-72.86	-18.55
GA	-46.35	-25.0	-32.32	-18.84

*a*: the 3D structure of HMG proteins was retrieved from the Protein Data Bank (www.rcsb.org) under the PDB codes 1HME (HMG box motif in the B domain of HMG1) and 2MRC (NMR Structure for High mobility group protein from *Plasmodium falciparum* 3D7). Docking experiments were performed as previously described;^(24)^ *b*: HMGB1 from *P. falciparum* and Box-B of Human HMGB1. Cpds: compounds; GA: glycyrrhetinic acid; GLR: glycyrrhizin; MGA: 3-O-mono-β-D-glucuronyl-glycyrrhetinic acid.

*18β-GA binding to Glyoxalase-1* - Glyoxalase 1 (GLO-1) is a glutathione-dependent enzyme which catalyses the conversion of toxic α-oxoaldehydes to non-toxic *D*-lactate. The mammalian enzyme is a dimeric zinc-containing metalloenzyme with two active sites located at the homodimer interface. It represents an ubiquitous detoxifying system, essential to eliminate methylglyoxal, but the enzyme also represents a potential target to treat Alzheimer’s disease, diabetes, cancer and other diseases. In 2015, it was reported that 18β-glycyrrhetinic acid (the 18β-epimer of GA) is a solid inhibitor of GLO-1, with a *K*i values of 0.29 μM (Fig. 4). The GA analogue carbenoxolone, bearing a succinate side chain in place of the carbohydrate moiety of GLR, is three times less active than GA.^18^ There is no data reported for GLR and MGA. Nevertheless, GA being an important active metabolite of GLR, this potential target must be taken into account, even more than the corresponding GLO-1 enzyme in *P. falciparum* (*Pf*GLO-1) has been considered as potential target to design antimalarial drugs.^25^ ^,^ ^26^ *Pf*GLO-1 is probably not the sole target of 18β-glycyrrhetinic acid. A molecular modeling study has suggested that this compound may bind to the *Plasmodium* lactate dehydrogenase enzyme (*Pf*LDH). It showed a moderate docking score of 71.2 for *Pf*LDH compared to chloroquine (LibDock docking score of 131.1) but apparently it would bind to the same NADH binding pocket of *Pf*LDH.^7^ This enzyme has been considered a promising molecular target for antimalarial drugs due to the parasite’s dependence on glycolysis for energy production.

**Fig. 4:**
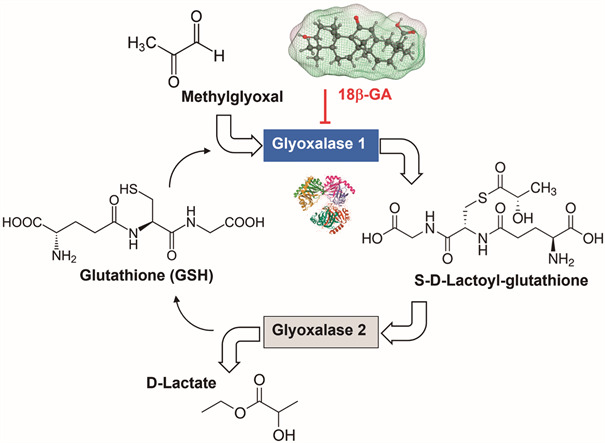
inhibition of Glyoxalase-1 by 18β-glycyrrhetinic acid (18β-GA), an enzyme implicated in the metabolism of toxic methylglyoxal converted into non-toxic *D*-lactate by the glyoxalase system.

*In conclusion* - The anti-malaria activity of GLR and its metabolites seems to rely essentially on two complementary activities: a membrane effect associated with the drug-induced disorganisation of lipid rafts and cholesterol sequestration, and the formation of stable complexes with HMGB1 proteins, including both human and *Plasmodium* HMGB1 (Fig. 5). In addition, the inhibition of the detoxifying enzyme glyoxalase 1 by the GLR metabolite glycyrrhetinic acid can be suggested, even if probably, this later activity little contributes to the global antimalaria activity of GLR. In contrast, the two other aspects are essential. Cholesterol-rich membrane microdomains, including lipid rafts, are known to be very important in the pathological process, to facilitate the entry of the parasite into cells.^27^ The disorganisation of lipid rafts, through cholesterol binding, could thus be a convenient process to limit host cell invasion. By trapping cholesterol, GLR can perturb and restrict the remodeling of human erythrocyte plasma membrane upon infection with the parasite. It is known that malaria parasites have the property to regulate the cholesterol contents in membranes and this action may affect raft structures and membrane trafficking.^28^ In addition to the membrane effects, GLR displays a major capacity to bind to HMG-box proteins, in particular to HMGB1. The sequestration of human HMGB1 by GLR, as well as its metabolite MGA, is certainly at the origin of the anti-inflammatory action of the drug, to limit the release of pro-inflammatory cytokines. Our analysis suggests that GLR could exert also a direct action of the HMGB1 protein from *P. falciparum*. The drug could thus inhibit the transcriptional activity of the parasite, thereby limiting its growth and invasion capacity.

**Fig. 5:**
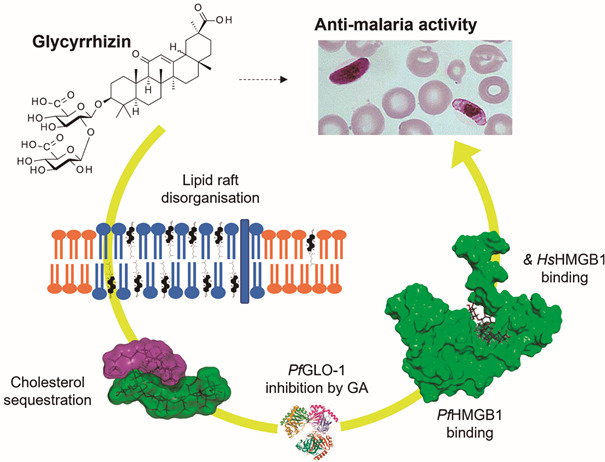
proposed anti-malaria mechanism of action of glycyrrhizin (GLR). The saponin drug disorganises the plasma membrane and cholesterol-rich lipid rafts, in part through the trapping of cholesterol, thus perturbing the cell entry of the parasite and its metabolism. In addition, GLR can block the activity of both *Plasmodium falciparum* HMGB1 (*Pf*HMGB1) and Human HMGB1 (*Hs*HMGB1), thus exerting an anti-inflammatory action and blocking HMGB1-related damage-associated molecular pattern molecule (DAMP) signals. Additionally, the potential inhibition of parasite glyoxalase-1 by GLR can be considered.

GLR displays antiplasmodial properties and a low toxicity to human red blood cells. It is one of the less hemolytic saponins.^11^ The limitation with GLR is not hemolysis but the risk of hypertension and hypokalemic-induced secondary disorders.^29^ However, these effects are manageable, and the drug is used, since a long time, in Asia for the treatment of liver diseases, without major apparent difficulties. It is also possible to modulate the drug structure, to predict the hemolytic potential and to combine GLR with other anti-malarial drugs.^30^ GLR is an amphipathic glycoside saponin that deserves further attention for the treatment of malaria, and other parasitic diseases.
